# An AIE Metal Iridium Complex: Photophysical Properties and Singlet Oxygen Generation Capacity

**DOI:** 10.3390/molecules28237914

**Published:** 2023-12-03

**Authors:** Weijin Zhu, Shengnan Liu, Ziwei Wang, Chunguang Shi, Qiaohua Zhang, Zihan Wu, Guangzhe Li, Dongxia Zhu

**Affiliations:** 1 Key Laboratory of Nanobiosensing and Nanobioanalysis at Universities of Jilin Province, Department of Chemistry, Northeast Normal University, 5268 Renmin Street, Changchun 130024, China; zhuwj727@nenu.edu.cn (W.Z.); liusn952@nenu.edu.cn (S.L.); wangzw026@nenu.edu.cn (Z.W.); shicg@nenu.edu.cn (C.S.); zhangqh899@nenu.edu.cn (Q.Z.); wuzihan20001009@163.com (Z.W.); 2 Jilin Provincial Science and Technology Innovation Center of Health Food of Chinese Medicine, Changchun University of Chinese Medicine, Changchun 130117, China

**Keywords:** aggregation-induced emission (AIE), iridium (III) complex, phosphorescence, singlet oxygen (^1^O_2_)

## Abstract

Photodynamic therapy (PDT) has garnered significant attention in the fields of cancer treatment and drug-resistant bacteria eradication due to its non-invasive nature and spatiotemporal controllability. Iridium complexes have captivated researchers owing to their tunable structure, exceptional optical properties, and substantial Stokes displacement. However, most of these complexes suffer from aggregation-induced quenching, leading to diminished luminous efficiency. In contrast to conventional photosensitizers, photosensitizers exhibiting aggregation-induced luminescence (AIE) properties retain the ability to generate a large number of reactive oxygen species when aggregated. To overcome these limitations, we designed and synthesized a novel iridium complex named **Ir-TPA** in this study. It incorporates quinoline triphenylamine cyclomethylated ligands that confer AIE characteristics for **Ir-TPA**. We systematically investigated the photophysical properties, AIE behavior, spectral features, and reactive oxygen generation capacity of **Ir-TPA**. The results demonstrate that **Ir-TPA** exhibits excellent optical properties with pronounced AIE phenomenon and robust capability for producing singlet oxygen species. This work not only introduces a new class of metal iridium complex photosensitizer with AIE attributes but also holds promise for achieving remarkable photodynamic therapeutic effects in future cellular experiments and biological studies.

## 1. Introduction

Photodynamic therapy is a selective process in which photosensitizer molecules absorb the appropriate wavelength of light and initiate the photoactivation process to produce toxic substances, thus leading to apoptosis or necrosis of pathological tissue cells [1,2]. Photodynamic therapy (PDT) has attracted extensive attention from researchers due to its advantages such as non-invasive, spatiotemporal controllability, and not easy to induce drug resistance [3,4,5]. The mechanism of photodynamic therapy is that under the corresponding wavelength of light, the photosensitizer in the ground state absorbs energy, transitions from the ground state to the single excited state, and then reaches the triple excited state through intersystem transition (ISC). The photosensitizer in the triple excited state returns to the ground state in two ways, corresponding to Type I and Type II photodynamic therapy processes, respectively [6]. In the process of Type I photodynamic therapy, photosensitizers in the triple excited state first undergo electron transfer with surrounding molecules to generate free radicals, and then interact with oxygen-containing substrates to produce reactive oxygen species such as hydroxyl free radicals (OH•), superoxide free radicals (O_2_•^−^), and hydrogen peroxide (H_2_O_2_), causing oxidative damage to cells [7]. In the process of type II photodynamic therapy, the photosensitizer in the triple excited state transfers energy to oxygen molecules in the process of returning to the ground state, forming singlet oxygen (^1^O_2_). Singlet oxygen has strong activity and can interact with numerous biological substrates to induce cell oxidation and then kill, thus achieving the purpose of photodynamic therapy [8,9]. ^1^O_2_ generated by Type II light reaction can oxidize major biomolecules of the nuclear membrane and cell membrane, such as unsaturated lipids and amino acids of proteins, and cause cell apoptosis, so ^1^O_2_ is toxic [10].

Although PDT possesses the aforementioned advantages, achieving enhanced clinical efficacy remains a significant challenge. In this regard, the precise selection and design of photosensitizers play a crucial role in determining the therapeutic outcomes of PDT [11]. The ability of traditional organic small molecules to transduce between systems has great limitations. Iridium’s large atomic number enables strong spin–orbit coupling, which is conducive to phosphor emission [12]. The excited states of iridium complexes are characterized not only by MLCT, but also by ligand-to-ligand charge transfer (LLCT) and in vivo charge transfer (ILCT) [13]. Iridium complexes, compared to other transition metal complexes, exhibit excellent photochemical and physical stability, large Stokes shift, and high intersystem crossover ability [14]. Consequently, they have emerged as extensively utilized transition metal complex materials across various domains such as bioimaging, electroluminescence, and photodynamic therapy [15,16,17,18,19]. Notably, iridium complexes have been extensively investigated as photosensitizers in photodynamic therapy [20,21]. For instance, we successfully synthesized UCNPs@Ir-2-N—a near-infrared absorbing photosensitizer—by combining an AIE iridium complex with upconversion nanoparticles (UCNPs). This study demonstrates the promising application potential of metal iridium complex PSs in PDT [22].

Traditional small molecule photosensitizers, such as porphyrins [23], usually have large planar conjugated structures and strong intermolecular π-π accumulation, and their luminous intensity will gradually weaken and even quench when they are aggregated, a phenomenon called Aggregation-caused Quenching (ACQ) [24]. This Aggregation-caused Quenching (ACQ) phenomenon significantly reduces their photosensitization ability, severely limiting their practical application [25,26]. In addition, the fluorescence emitted by conventional photosensitizers is usually non-luminous, resulting in reduced imaging sensitivity [27,28] and placing great limitations on the clinical application of many photosensitizers in photodynamic therapy (PDT). In 2001, academician Tang’s research group found the opposite phenomenon to ACQ: under dilute solution conditions, these photosensitizers emit weak or even no light, but with the increase in concentration, the luminescence is significantly enhanced [29]. Unlike ACQ photosensitizers, photosensitizers with Aggregation-induced Emission (AIE) properties typically have a non-planar structure. The intramolecular motion in the aggregation state is limited; thus, the rigidity of the molecular structure is increased, the non-radiative attenuation pathway is reduced, and, finally, the radiative attenuation pathway dominates. Limited intramolecular motion reduces non-radiative energy dissipation while enhancing fluorescence emission and sensitizing a large number of reactive oxygen species (ROS) in the aggregation state, thus providing a huge advantage for image-guided PDT [30,31,32]. However, there are few examples of iridium complexes with AIE properties used in photodynamic therapy. Therefore, the development of iridium-based AIE active photosensitizers is of great significance to promote the application of PDT.

**Scheme 1:**
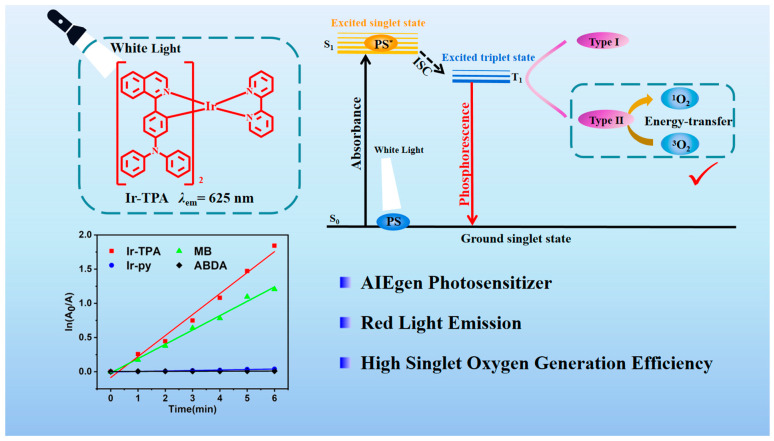
Structure of **Ir-TPA** and schematic diagram of **Ir-TPA** producing singlet oxygen. * Represents an excited state and the checkmark indicates that the work is a Type II photodynamic process.

In this study, we developed and synthesized an iridium complex called **Ir-TPA** by incorporating a quinoline triphenylamine ligand. We conducted comprehensive investigations on the photophysical properties, AIE characteristics, spectral properties, and singlet oxygen generation capacity of **Ir-TPA**. As shown in Scheme 1, our findings demonstrate that **Ir-TPA** exhibits excellent optical properties, displays remarkable AIE behavior, and possesses strong capability in generating reactive oxygen species. This research confirms the significant potential of AIE properties in enhancing the effectiveness of photodynamic therapy and presents a fresh perspective on utilizing iridium complexes in the field of photodynamic therapy.

## 2. Results and Discussion

### 2.1. Analysis of Photophysical Properties of Complexes

**Figure 1:**
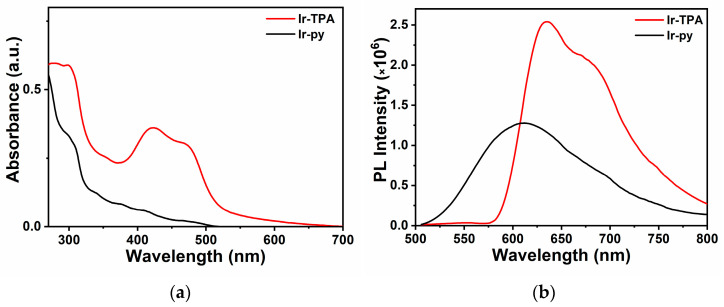
(**a**) Uv absorption spectra of **Ir-TPA** ^1^, **Ir-py** ^2^ (10^−5^ M) in CH_3_CN/H_2_O (*V*/*V* = 1/1); (**b**) fluorescence emission spectra, ^1^ *λ*_ex_ = 467 nm ^2^ *λ*_ex_ = 395 nm.

The photophysical properties of **Ir-TPA** and **Ir-py** were analyzed by ultraviolet absorption and fluorescence emission spectra. As shown in Figure 1, similar to the iridium complexes reported in the literature, **Ir-TPA** has two characteristic absorption peaks in a certain wavelength range. There is a strong characteristic absorption peak in the wavelength range of 250~350 nm, which is caused by the central charge (^1^LC, π-π*) transition of the ligand in the metal iridium complex. There is a weak absorption band in the 350 nm to visible light band, which can be attributed to metal–ligand charge transfer transition (MLCT) and ligand–ligand charge transfer (LLCT) of the metal iridium complex [33]. It can be clearly seen from the figure that the molar absorption coefficient of **Ir-TPA** was significantly enhanced compared with **Ir-py** after the introduction of the quinoline triphenylamine ligand (Table 1). Especially in the 425 nm visible region, the molar coefficient of **Ir-TPA** is 41,680 m^−1^cm^−1^, which is eight times that of **Ir-py**. It can be shown that the absorption peak of the iridium complex is obviously enhanced after the introduction of the quinoline triphenylamine ligand. Subsequently, we determined the photoluminescent quantum yields (PLQYs) and excited state lifetimes of **Ir-TPA** and **Ir-py** at CH_3_CN/H_2_O (*v*/*v* = 1/9) and summarized the corresponding photophysical data in Table 1. It can be seen from the data in the table that **Ir-TPA** and **Ir-py** have good photophysical properties.

### 2.2. Analysis of AIE Properties of Complexes

**Figure 2:**
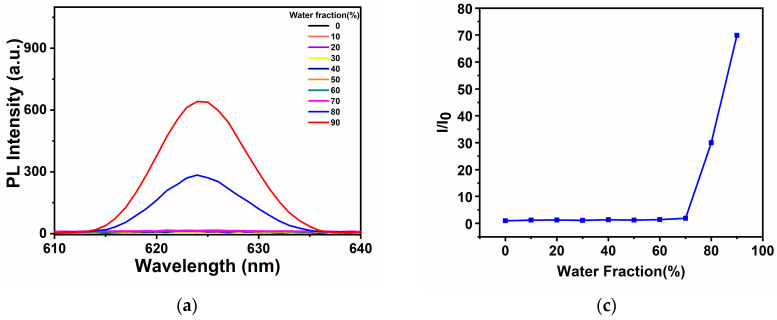

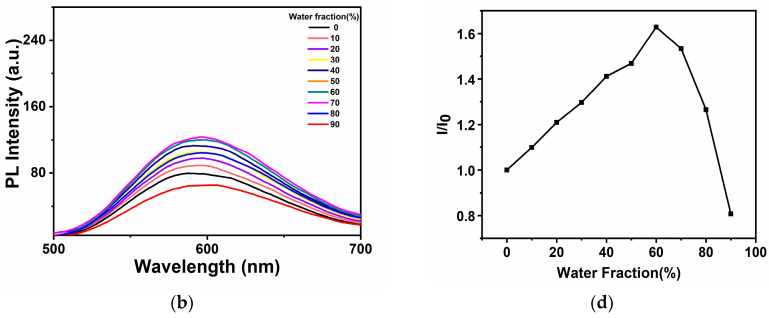
(**a**) **Ir-TPA** ^1^, (**b**) **Ir-py** ^2^ fluorescence emission spectra in different proportions of CH_3_CN/H_2_O, I/I_0_ value image of (**c**) **Ir-TPA**, (**d**) **Ir-py**. ^1^ *λ*_ex_ = 467 nm ^2^ *λ*_ex_ = 395 nm.

The AIE properties of **Ir-TPA** and **Ir-py** were analyzed in CH_3_CN and water mixed system solution (water content ranged from 0 to 90%). As shown in Figure 2, **Ir-py** exhibited weak emission in pure CH_3_CN solution, but with the increase in water content, the luminescence gradually weakened, showing obvious aggregation-induced quenching phenomenon. Iridium complex **Ir-TPA** was obtained by using quinoline triphenylamine derivatives as cyclometalated ligands, which basically did not emit light in pure CH_3_CN. The luminescence of **Ir-TPA** increased gradually with the increase in the water content of the bad solvent. When the water content of the mixed solution reaches 80%, **Ir-TPA** emits bright red light, and **Ir-TPA** has typical AIE characteristics.

**Ir-TPA** showed more emission redshift than **Ir-py** due to the better conjugation of cyclometal ligands derived from quinoline triphenylamine. **Ir-TPA** exhibits excellent red light emission and larger Stokes shifts value, indicating that in future biological experiments, autofluorescence interference will be effectively avoided, and the signal-to-noise ratio of imaging will be improved, which has a broad prospect in future biological applications.

**Figure 3:**
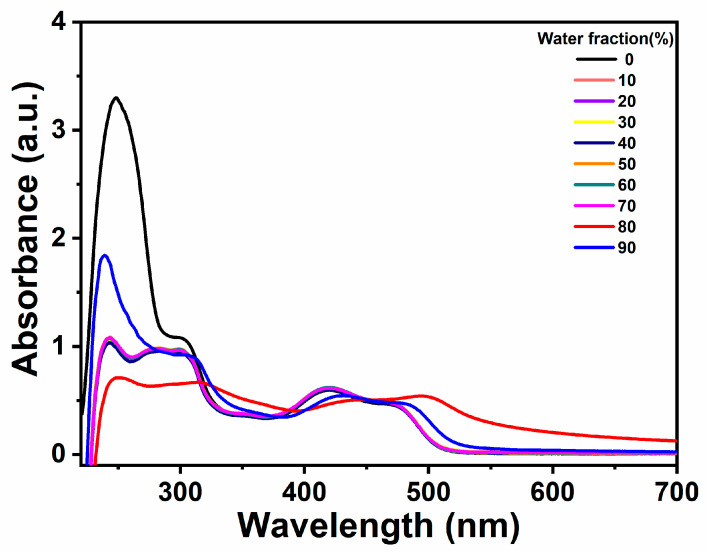
Ultraviolet–visible absorption spectra of **Ir-TPA** (10^−5^ M) in CH_3_CN and H_2_O mixed solutions.

Then, we further investigated the AIE phenomena of **Ir-TPA** by UV-Vis absorption spectra. As can be seen from Figure 3, with the gradual increase in water content in the system, the tail of the absorption spectrum slightly warped, indicating that the Mi scattering phenomenon occurred [34].

**Figure 4:**
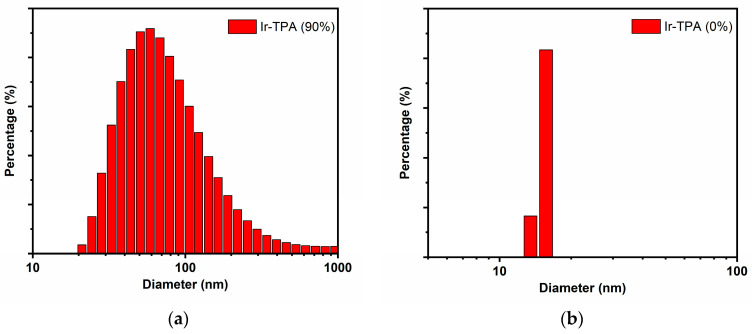
DLS particle size map of **Ir-TPA** at room temperature in (**a**) CH_3_CN/H_2_O (*V/V* = 1/9) and (**b**) CH_3_CN solutions.

As shown in Figure 4, we further used dynamic light scattering (DLS) to analyze the hydrated particle size of **Ir-TPA** in CH_3_CN/H_2_O (*v*/*v* = 1/9) and CH_3_CN solutions, respectively, to prove that **Ir-TPA** will aggregate when the proportion of poor solvent water increases. DLS showed that the average hydration kinetic radius of **Ir-TPA** at 0% (CH_3_CN solution) and 90% (CH_3_CN/H_2_O = 1/9) water content was 15.33 nm and 66.27 nm, respectively. The average hydration kinetic radius of **Ir-TPA** is more than four times that of the dispersion state when the content of the poor solvent water reaches 90%. Therefore, it can be well explained that **Ir-TPA** does occur as an aggregation phenomenon. Through the above experimental results, we can conclude that the complex **Ir-TPA** has good AIE performance, which is due to the emission enhancement caused by aggregation in poor solvents.

### 2.3. Analysis of Singlet Oxygen Generation Capacity in Solution

**Figure 5:**
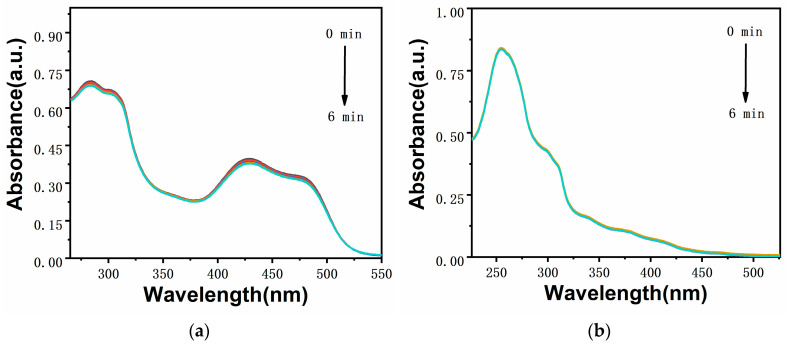
UV absorption spectra of (**a**) Ir-TPA; (**b**) Ir-py (25µM) at white light (400–700 nm, 20 mW cm^−2^) for 0–6 min.

**Figure 6:**
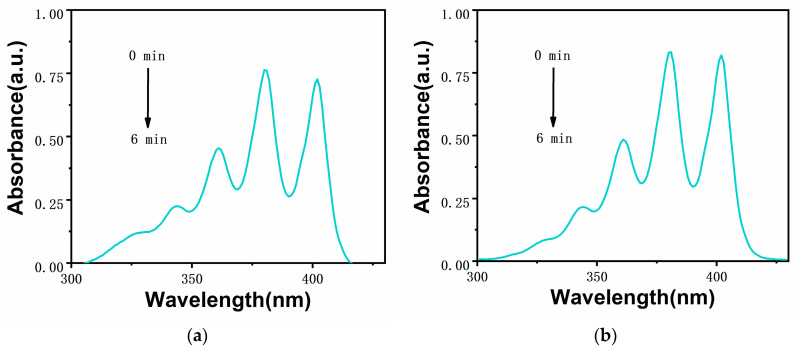
Ultraviolet–visible absorption spectra of ABDA (30 µg·mL^−1^) without light in the presence of (**a**) **Ir-TPA**; (**b**) **Ir-py** (25 µM).

**Figure 7:**
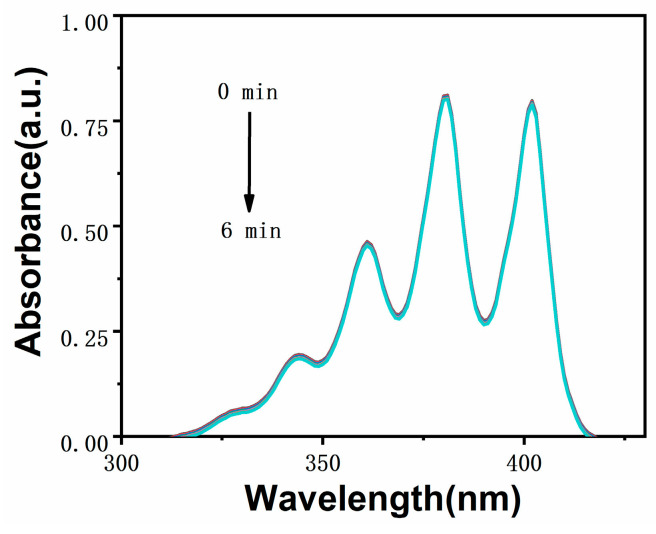
Ultraviolet–visible absorption spectra of ABDA under white light (400–700 nm, 20 mW cm^−2^) for 0–6 min.

**Figure 8:**
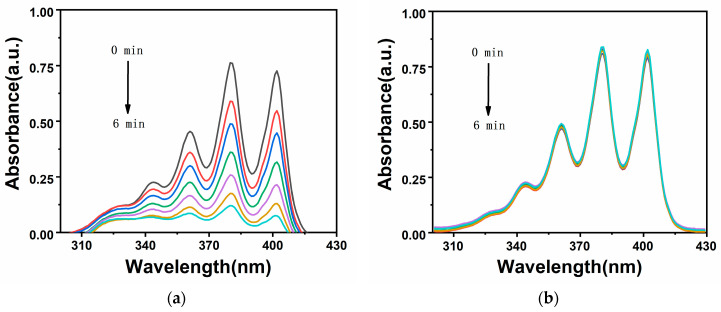
Ultraviolet–visible absorption spectra of ABDA (30 µg·mL^−1^) under the irradiation of a white light LED lamp (400–700 nm, 20 mW cm^−2^) in the presence of (**a**) **Ir-TPA**; (**b**) **Ir-py** (25 µM) for 0–6 min.

**Figure 9:**
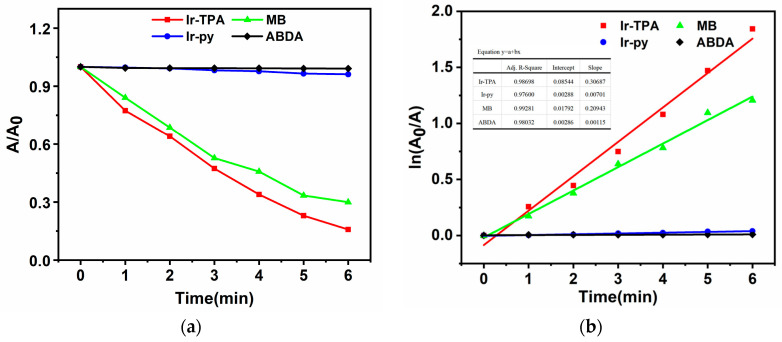
In the presence of **Ir-TPA**/**Ir-py**/MB (25 µM), under the condition of illumination (400–700 nm, 20 mW cm^−2^), (**a**) the radiation attenuation curve of ABDA at 380 nm at different times; (**b**) time-dependent generation kinetics curve of ^1^O_2._

The ability of photosensitizer to produce singlet oxygen is very important for the effect of photodynamic therapy. The singlet oxygen production capacity of two iridium complexes **Ir-TPA** and **Ir-py** at CH_3_CN:H_2_O (*V:V* = 1:9) was evaluated by monitoring the absorbance change of ABDA at 380 nm using ABDA as an indicator. As shown in Figure 5, Figure 6 and Figure 7, for (1) the light group containing PSs; (2) the unilluminated group containing PSs and ABDA; and (3) for the ABDA light group alone, the absorption intensity basically did not change during the time of illumination 360 s, which proves that **Ir-TPA** and **Ir-py** have good light stability. As shown in Figure 8, when irradiated with a white LED lamp, the absorption of **Ir-TPA** and **Ir-py** at 380 nm is significantly reduced, which proves the production of singlet oxygen under the illumination condition. As shown in Figure 9, the singlet oxygen generation capacity of **Ir-TPA** and **Ir-py** both conform to the first-order kinetic equation. The higher the slope, the stronger the singlet oxygen generation capacity, and the singlet oxygen generation efficiency is **Ir-TPA** > Methylene Blue (MB) > **Ir-py**. As shown in Table 2, using methylene blue as the reference, the ^1^O_2_ quantum yield of **Ir-TPA** is as high as 83.5%, indicating that **Ir-TPA** can efficiently produce singlet oxygen, and they will play an important role in the application of PDT cancer as Ps.

## 3. Materials and Methods

### 3.1. General Information

Reagents and solvents should be used as received from the supplier. Unless otherwise specified, all purification is performed using 200–300 mesh silica gel from the supplier by column chromatography. The structure was confirmed by Bruker AV600 NMR spectrometer (Bruker, Billerica, MA, USA) and Bruker autoFlex III mass spectrometer (Bruker, Billerica, MA, USA). A Shimadzu UV-3100 spectrophotometer (Shimadzu, Kyoto, Japan) was used for ultraviolet–visible experiments. The fluorescence emission spectra were measured by Edinburgh FLS920 steady-state transient fluorescence spectrometer (Edinburgh Instruments Ltd., Livingston, UK). Dynamic light scattering (DLS) was tested using NanoZS90.

### 3.2. Solution Preparation Method

The spare solution of **Ir-TPA** and **Ir-py** (1 mM) was prepared in acetonitrile. The preparation method of the test solution was as follows: during the test, 30 μL **Ir-TPA** and **Ir-py** reserve solution, 270 μL acetonitrile, and 2700 μL ultra-pure water were prepared into the test solution with a total volume of 3 mL.

### 3.3. Fluorescent Spectrum Test Method

The steady-state spectra PL and phosphor decay lifetime under photoluminescence were measured by an Edinburgh FLS920 steady-state transient fluorescence spectrometer, and the quantum yield ΦPL was measured by integrating a sphere with acetonitrile/water (*v*/*v* = 1/9) as the basis. A 450 W xenon lamp is used as the light source when measuring the attenuation life of the spectrum.

### 3.4. Test Method for Singlet Oxygen in Solution

The efficacy of photodynamic therapy is evaluated by the level of singlet oxygen production in the solution. The ^1^O_2_ formation capacity of **Ir-TPA**/**Ir-py** at CH_3_CN/H_2_O = 1/9 was evaluated using 9, 10-anthracenedi-bis (methylene) dicarboxylic acid (ABDA) as an indicator. After a certain time of light irradiation, the absorbance of ABDA at 380 nm was significantly reduced, indicating that the photosensitizer sensitized oxygen to produce ^1^O_2_. In this experiment, CH_3_CN/H_2_O = 1/9 solution containing **Ir-TPA**/**Ir-py** (25 µM) was prepared and mixed with ABDA (30 µg·mL^−1^), and then exposed to white light (400–700 nm, 20 mW cm^−2^) for irradiation. The absorption intensity of ABDA at 380 nm was monitored every 60 s.

### 3.5. Synthesis and Characterization of Complexes

**Figure 10:**
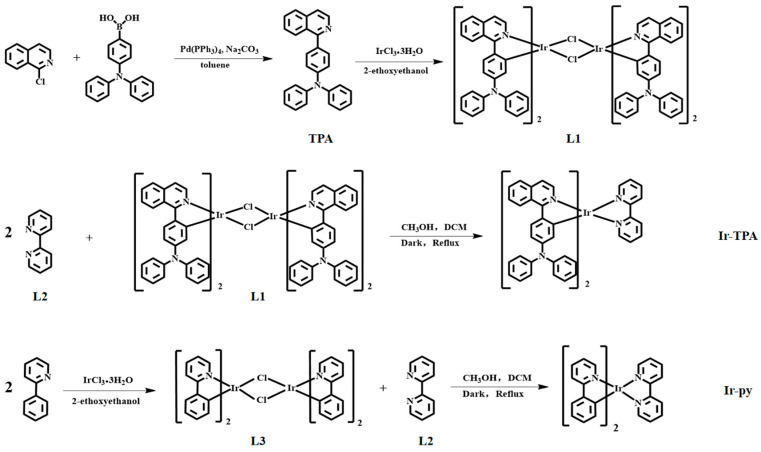
Iridium (III) complex synthesis route.

The resultant route is detailed in Figure 10.

#### 3.5.1. Synthesis of Cyclometalated Ligand **TPA**

Trianiline 4-borate (0.972 g, 3.36 mmol) and 1-chloroisoquinoline (0.496 g, 3.06 mmol) were dissolved in 30 mL toluene, and the catalyst tetrtriphenylphosphine palladium (0.177 g, 0.15 mmol) and 2 mol/L sodium carbonate solution 20 mL were added. The reaction was kept at 110 °C for 48 h in a deoxygenated environment. After the reaction, the organic phase was extracted and dried with anhydrous magnesium sulfate, filtered, and purified by silica gel column chromatography (dichloromethane/petroleum ether, 10/5). The product is a light yellow solid with a yield of 68%. ^1^H NMR (500 MHz, CDCl_3_) δ 8.59 (d, *J* = 5.7 Hz, 1H), 8.23 (d, *J* = 8.5 Hz, 1H), 7.87 (d, *J* = 8.2 Hz, 1H), 7.69 (t, *J* = 7.2 Hz, 1H), 7.62–7.59 (m, 3H), 7.56 (t, *J* = 7.7 Hz, 1H), 7.29 (t, *J* = 7.9 Hz, 4H), 7.21 (dd, *J* = 13.3, 8.1 Hz, 6H), and 7.05 (d, *J* = 7.3 Hz, 2H).

#### 3.5.2. Synthesis of **L1**

**TPA** (0.930 g, 2.5 mmol) and IrCl_3_·3H_2_O (0.317 g, 1 mmol) were dissolved in a mixed solution of 30 mL ethylene glycol ether and 10 mL water, and the reaction was continued for 24 h at 120 °C under nitrogen atmosphere. At the end of the reaction, water was added to the round-bottom flask and continued to stir for 30 min. The Brinell funnel was used for pumping and filtering, and the obtained solids were put into the oven for 24 h. After drying, the product became a deep red solid with a yield of 80%. The product is directly used in subsequent reactions.

#### 3.5.3. Synthesis of **Ir-TPA**

**L1** (0.194 g, 0.1 mmol), **L2** (0.0312 g, 0.2 mmol), methanol 20 mL, and dichloromethane 20 mL were added as mixed solvents in single-mouth bottles, respectively. N_2_ was charged for protection under dark conditions, and reflux was carried out at 78 °C for 8 h. At the end of the reaction, solid potassium hexafluorophosphate (1 mmol, 0.184 g) was added to the bottle, stirring for 45 min, and then the solvent was filtered and spun dry. The solid is dissolved in a small amount of dichloromethane, and the precipitation is obtained by filtration after reverse precipitation with petroleum ether. The crude product was purified by silica gel column chromatography (methylene chloride/acetone, 10/8). The product is a dark red solid with a yield of 35%. ^1^H NMR (600 MHz, CDCl_3_) δ 8.74–8.72 (m, 1H), 8.66 (d, *J* = 8.2 Hz, 1H), 8.14 (t, *J* = 7.8 Hz, 1H), 8.02 (d, *J* = 8.9 Hz, 1H), 7.88 (d, *J* = 5.2 Hz, 1H), 7.66 (t, *J* = 4.9 Hz, 3H), 7.46–7.44 (m, 1H), 6.98 (dd, *J* = 13.5, 6.8 Hz, 6H), 6.92 (d, *J* = 7.9 Hz, 4H), 6.81 (t, *J* = 6.6 Hz, 3H), and 6.76 (dd, *J* = 8.8, 2.3 Hz, 1H). ESI-MS: [*m*/*z*] = 1091.3508 (calcd: 1091.35).

#### 3.5.4. Synthesis of **Ir-py**

We dissolved **L3** (0.1608 g, 0.1 mmol) and **L2** (0.0312 g, 0.2 mmol), methanol 20 mL, and dichloromethane 20 mL as mixed solvents in a single mouth bottle. N_2_ was charged for protection under dark conditions, and reflux was carried out at 78 °C for 8 h. At the end of the reaction, solid potassium hexafluorophosphate (1 mmol, 0.184 g) was added to the bottle, stirred for 45 min, and then the solvent was filtered and spun dry. The solid is dissolved in a small amount of dichloromethane, and the precipitation is obtained by filtration after reverse precipitation with petroleum ether. The yellow solid was obtained after drying, and the yield was 44%. ^1^H NMR (600 MHz, DMSO) δ 8.89 (d, *J* = 8.2 Hz, 1H), 8.28 (t, *J* = 7.2 Hz, 2H), 7.95–7.91 (m, 2H), 7.88 (d, *J* = 5.3 Hz, 1H), 7.72–7.69 (m, 1H), 7.62 (d, *J* = 5.7 Hz, 1H), 7.16 (t, *J* = 6.6 Hz, 1H), 7.03 (t, *J* = 7.5 Hz, 1H), 6.91 (t, *J* = 7.4 Hz, 1H), and 6.20 (d, *J* = 7.6 Hz, 1H). ESI-MS: [*m*/*z*] = 657.1636 (calcd: 657.16).

## 4. Conclusions

The iridium metal complex **Ir-TPA**, possessing aggregation-induced emission (AIE) properties, was designed and synthesized in this study. By incorporating quinoline triphenylamine metallized ligands, the AIE properties of **Ir-TPA** were enhanced, leading to a significant improvement in the complex’s absorption capacity. Notably, the complex exhibited red light emission with a maximum wavelength of 625 nm. In-depth investigations were conducted on the photophysical properties, AIE characteristics, spectral features, and reactive oxygen generation capability of **Ir-TPA**. The results demonstrated that **Ir-TPA** displayed excellent optical properties along with pronounced AIE behavior and strong reactive oxygen species production ability. This work validates the substantial potential of AIE properties in enhancing photodynamic therapy efficacy and provides novel insights into the application of iridium complexes in this field.

## Figures and Tables

**Table 1:** Photophysical data of **Ir-TPA** and **Ir-py**.

	*Λ*_abs_ (nm)	*λ*_em_ (nm)	Ф_p_ (%)	Τ_p_ (ns)	Ɛ (m^−1^·cm^−1^)
**Ir-TPA** ^1^	300; 425; 470	625	16.34	218.6	62,513; 41,680; 34,300
**Ir-py** ^2^	310	590	4.29	69.5	30,247

^1^ Determined at CH_3_CN/H_2_O (*v*/*v* = 1/9) with a concentration of 1.0 × 10^−5^ M, *λ*_ex_ = 467 nm. ^2^ Determined at CH_3_CN/H_2_O (*v*/*v* = 1/9) with a concentration of 1.0 × 10^−5^ M, *λ*_ex_ = 395 nm.

**Table 2:** Singlet oxygen generation efficiency data of **Ir-TPA, Ir-py,** and MB.

	Linear Fit Slope	Intercept	Singlet Oxygen Yield (%)
**Ir-TPA**	0.30687	0.08544	83.5
**Ir-py**	0.00701	0.00288	24.0
MB	0.20943	0.01792	52.0

## Data Availability

All data generated or analyzed during this study are included in this published article and its Supplementary Information Files.

## Supplementary Materials

The following supporting information can be downloaded at: https://www.mdpi.com/article/10.3390/molecules28237914/s1.
