# Supplementary material for: An AIE Metal Iridium Complex: Photophysical Properties and Singlet Oxygen Generation Capacity
Source: Molecules. 2023 Dec 3;28(23):7914. doi: 10.3390/molecules28237914 (PMC10708252; doi:10.3390/molecules28237914)
Supplement: Supplementary file 1 [file molecules-28-07914-s001.zip › molecules-2727194-supplementary.pdf]

Supporting information

An AIE Metal Iridium Complex: Photophysical Properties and Singlet Oxygen Generation Capacity

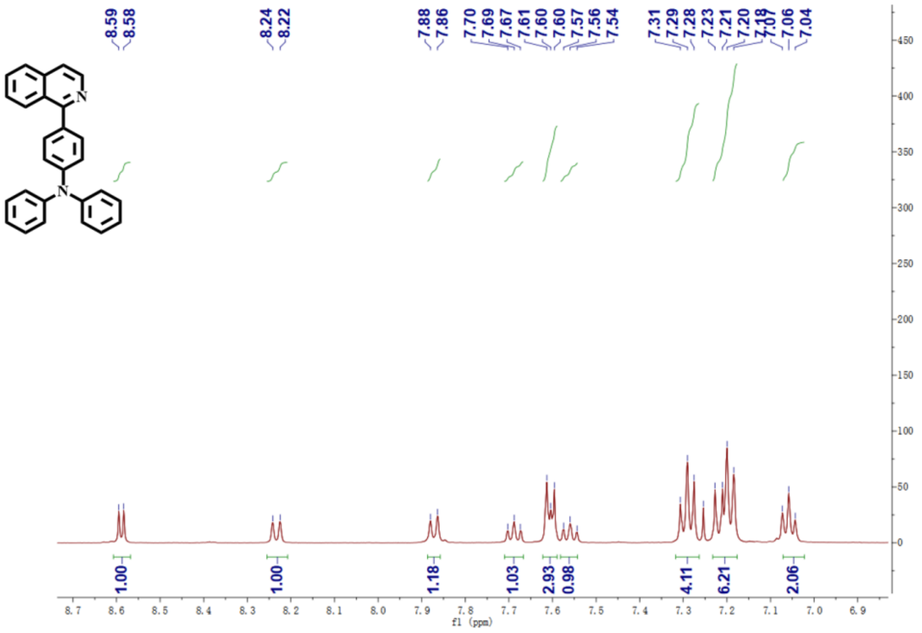

Figure S1. ^{1}H NMR nuclear magnetic spectrum of **TPA** in $CDCl_3$

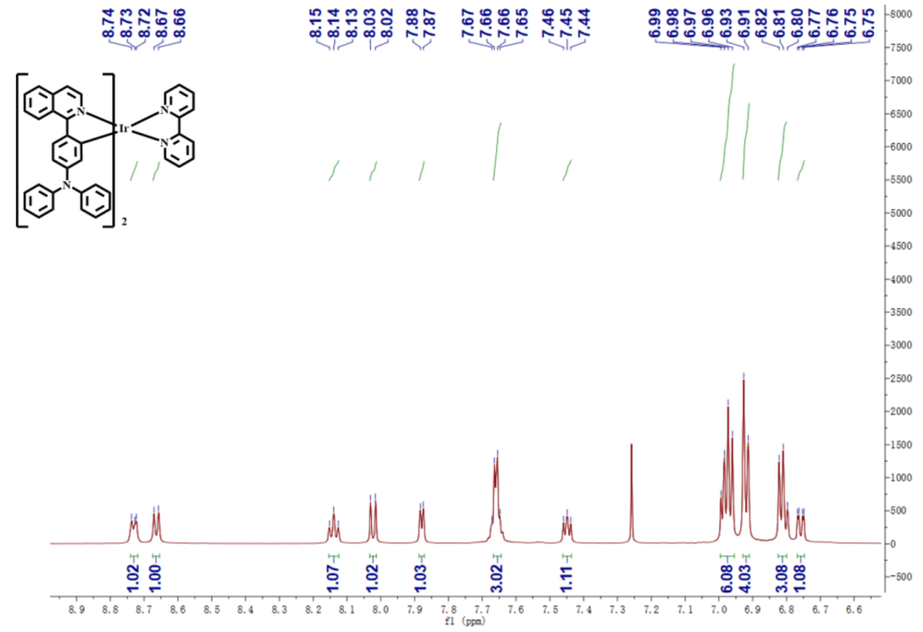

Figure S2. ^{1}H NMR nuclear magnetic spectrum of **Ir-TPA** in $CDCl_3$

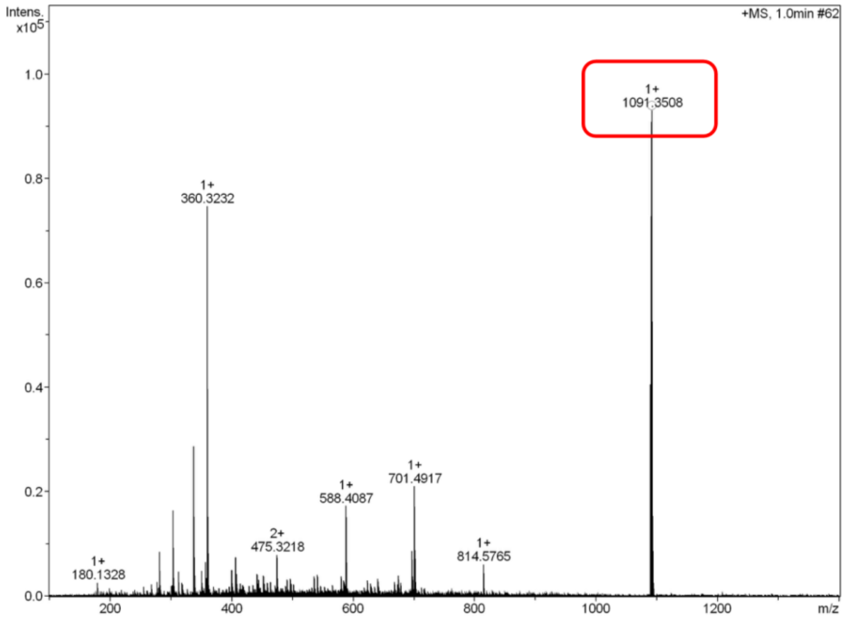

Figure S3. Mass spectrum of the complex **Ir-TPA**

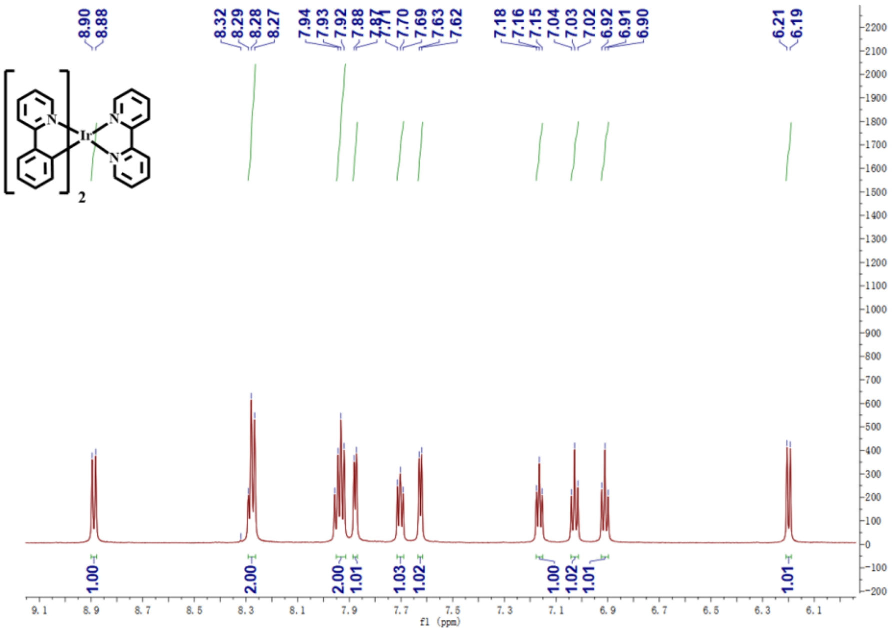

Figure S4. ^{1}H NMR nuclear magnetic spectrum of **Ir-py** in DMSO

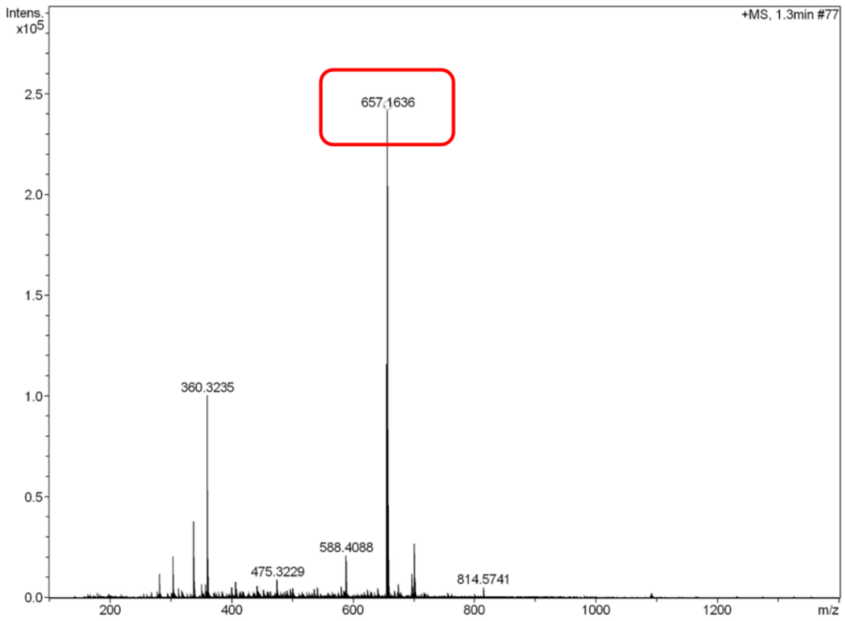

Figure S5. Mass spectrum of the complex **Ir-py**
